# Unraveling Causal Mechanisms of Top-Down and Bottom-Up Visuospatial Attention with Non-invasive Brain Stimulation

**DOI:** 10.1007/s41745-017-0046-0

**Published:** 2017-12-06

**Authors:** Sanjna Banerjee, Shrey Grover, Devarajan Sridharan

**Affiliations:** Centre for Neuroscience, Indian Institute of Science, Bangalore, 560012 India

## Abstract

Attention is a process of selection that allows us to intelligently navigate the abundance of information in our world. Attention can be either directed voluntarily based on internal goals—“top-down” or goal-directed attention—or captured automatically, by salient stimuli—“bottom-up” or stimulus-driven attention. Do these two modes of attention control arise from same or different brain circuits? Do they share similar or distinct neural mechanisms? In this review, we explore this dichotomy between the neural bases of top-down and bottom-up attention control, with a special emphasis on insights gained from non-invasive neurostimulation techniques, specifically, transcranial magnetic stimulation (TMS). TMS enables spatially focal and temporally precise manipulation of brain activity. We explore a significant literature devoted to investigating the role of fronto-parietal brain regions in top-down and bottom-up attention with TMS, and highlight key areas of convergence and debate. We also discuss recent advances in combinatorial paradigms that combine TMS with other imaging modalities, such as functional magnetic resonance imaging or electroencephalography. These paradigms are beginning to bridge essential gaps in our understanding of the neural pathways by which TMS affects behavior, and will prove invaluable for unraveling mechanisms of attention control, both in health and in disease.

## Introduction

The world around us inundates our senses with an overabundance of information. Yet, our capacity to process and act on this information is limited. Attention is the remarkable cognitive process that enables us to select and prioritize the processing of the most relevant stimuli for guiding behavior. Attention can be directed to stimuli at specific locations in space (spatial attention) or to specific features of stimuli (feature-based attention). In each case, attention can be directed with or without concomitant shifts of gaze toward the attended stimulus (overt and covert attention, respectively). Finally, attention may be directed voluntarily (endogenous or top-down) or captured automatically (bottom-up or exogenous).

Here, we seek to review the current state of knowledge on the neural basis of visual spatial attention, specifically exploring the dichotomy between top-down and bottom-up control of attention. Top-down attention and bottom-up attention

**Top-down attention:** Voluntary deployment of attention due to internal goals or task demands. Also known as endogenous or goal-directed attention.

**Bottom-up attention:** Automatic capture of attention by salient stimuli or events in the world. Also known as exogenous or stimulus-driven attention.

produce largely similar effects on behavior, but also exhibit some important differences (reviewed in Sect. 3.1). The neural basis of these similarities and differences has been investigated with a variety of techniques, including single- and multi-unit electrophysiology, and imaging techniques like functional magnetic resonance imaging (fMRI)

**fMRI:** functional magnetic resonance imaging—a non-invasive technique for recording, with high spatial resolution, brain activity by measuring associated blood flow changes.,

magnetoencephalography (MEG) and electroencephalography (EEG) (reviewed in Sect. 3.2). These investigations have revealed key functional brain networks and electrophysiological markers that are characteristic of each mode of attention. However, imaging or recording studies cannot demonstrate a causal role of these brain networks and electrical activity patterns in attention.

Non-invasive neurostimulation technologies, on the other hand, offer the ability to investigate the causal link between brain and behavior (reviewed in Sect. 2). We discuss, specifically, novel insights provided by transcranial magnetic stimulation (TMS)

**TMS:** Transcranial magnetic stimulation—a non-invasive technique to activate or suppress neural activity by inducing electric currents with focally applied magnetic pulses.

into mechanisms of top-down and bottom-up attention (reviewed in Sect. 4). Powerful, emerging technologies, that combine neurostimulation with concurrent brain recordings (EEG/fMRI), have the potential to provide a more precise picture of these attention mechanisms (reviewed in Sect. 5). We conclude by discussing key challenges (Sect. 6) and the future outlook (Sect. 7) for non-invasive brain stimulation in understanding how attention works in the brain, both in health and in disease.

## Principles of Neurostimulation

Neurostimulation involves altering the electrical activity of the brain, and can be applied either invasively or non-invasively. The use of invasive neurostimulation is well established in clinical interventions like deep brain stimulation of the basal ganglia for the treatment of Parkinson’s disease symptoms, vagus nerve stimulation for treatment of epilepsy, transcutaneous nerve stimulation for neuropathic pain, and the like.^1^^–^ 3 Non-invasive neurostimulation techniques are a relatively recent development. For cognitive neuroscientists, non-invasive neurostimulation techniques offer great potential for probing the causal roles of different brain areas in orchestrating cognitive processes in the healthy human brain. These techniques include, primarily, electromagnetic methods such as transcranial magnetic stimulation (TMS) and transcranial electrical stimulation (tES)

**tES:** Transcranial electrical stimulation—a non-invasive technique to modulate neural activity by direct application of currents to the surface of the scalp..

Each of these techniques affects neural activity by influencing the excitability of neuronal populations in the stimulated region.

tES involves electrical stimulation applied on the scalp surface, either in the form of direct current (tDCS) or alternating current (tACS)

**tDCS/tACS:** Transcranial direct/alternating current stimulation, used to stimulate the brain with steady or oscillatory electric currents..

The electric fields generated can alter neuronal activity and cortical excitability in the area of the brain stimulated; these changes are reversible depending on the strength and duration of stimulation.^4^ tDCS involves applying a constant current for a few minutes to depolarize or hyperpolarize underlying neural tissue.^4^ It is commonly held that positive (anodal) stimulation results in local depolarization and increases neuronal excitability, whereas negative (cathodal) stimulation leads to hyperpolarization^4^ (but see 5 ^,^ 6). tACS, on the other hand, involves applying time-dependent oscillatory (e.g., sinusoidal or square waves) currents at specific frequencies, and can be used to entrain brain oscillations at specific frequencies.^7^^,^ 8 Despite its simplicity, a key disadvantage of tES is the lack of spatial focality: neural activity at distal sites, up to several centimeters from the electrodes, may be modulated by the applied currents^9^ (but see^7^).

In contrast to tES, TMS can be used for spatially focal (and temporally precise) manipulations of neural activity. Originally developed by Barker et al.^10^ for testing corticospinal integrity in clinical patients, its use in cognitive research increased as the use of stimulation methods involving scalp currents fell out of favor due to the diffuse and discomforting nature of direct electrical stimulation. TMS works on the principle of electromagnetic induction. It utilizes the magnetic field produced by brief current pulses through a stimulating coil held tangential to the site of stimulation. This time-varying magnetic field crosses through the scalp and induces a current in brain tissue parallel to the coil, without activating pain fibers of the scalp.^11^^,^ 12 With optimized designs of standard figure-of-eight coils, the area of tissue stimulated is expected to be approximately 4 cm square with a depth of stimulation up to approximately 2 cm through the cortex.^13^ In addition, as there is no scalp diffusion of current, TMS results in stronger, more effective stimulation as compared to tES (where stimulation is applied to the scalp surface).

TMS can be delivered in the form of single, isolated pulses or as temporally patterned bursts. Early studies used single-pulse TMS on mainly motor and sensory cortices to delineate cortical motor/sensory maps, or for clinical tests.^14^^–^ 18 By the 90s, advancement in stimulator design allowed the efficient use of a more protracted stimulation protocol called repetitive TMS or rTMS

**rTMS:** repetitive TMS by applying a train of magnetic pulses, widely used to enhance the effects of TMS.,

involving the delivery of trains of multiple pulses^18^^–^ 20 delivered at a fixed frequency as well as with combinations of slow and fast frequencies (e.g., theta burst stimulation). The therapeutic effects of rTMS were found to be more robust than single-pulse TMS.^21^ Since then, both low-frequency (up to 1 Hz) and high-frequency (up to 60 Hz) rTMS have been used to probe the roles of the prefrontal, parietal, temporal and occipital cortex in various cognitive processes, including visual perception, attention, working memory, lateralization of language, semantic coding, decision making, and the like.^11^^,^ 22 ^–^ 26 In addition, rhythmic rTMS has been applied to entrain naturally occurring neural oscillations, to understand the causal role of these oscillations in various cognitive processes (see Sect. 4).

Investigating the precise neurophysiological mechanisms of TMS is an active area of research.^27^^–^ 30 The time-varying magnetic field produced by the coil generates eddy currents in the brain tissue through electromagnetic induction. This in turn gives rise to a spatially varying electric field within the tissue, the spread and direction of which depends upon the intensity of the pulse and the shape of the coil. A specific, directed component of this spatially varying field influences the electrical gradient across the membrane (the membrane potential); this influence is maximal in areas with the strongest induced electric field, and in fiber bends and branches.^27^ This rapid change in membrane potential may have many effects on the neuron’s state, including increasing the neuron’s excitability by raising its resting potential, triggering immediate action potentials, or changing its long-term response dynamics.^31^ A particular brain region may often contain different neuronal subtypes, and each subtype may be differently oriented in the tissue or possess unique membrane properties. Thus, the overall effect of a particular TMS protocol over a region is the combination of all the responses of its constituent neuronal subpopulations.^26^^,^ 31 For instance, single-pulse TMS over the motor cortex has been shown to affect both pyramidal neurons and inter-neurons, producing specific potential change patterns called D-waves (direct waves) and I-waves (indirect waves), respectively.^30^ Apart from immediate electrophysiological changes induced by single pulses, repeated stimulation may induce short-term neuroplastic changes, which produce sustained effects that outlast the stimulation. Pharmacological investigations implicate induction of NMDA (N-methyl-D-aspartate) receptor-based synaptic plasticity as a potential mechanism for the sustained effects of theta burst stimulation in humans,^32^ with similar mechanisms observed in rats during low-frequency rTMS.^33^

Empirical evidence in humans indicates that the exact effect of TMS—facilitation or suppression—depends on the exact stimulation protocol followed. The effects depends crucially on the number and temporal order of pulses,^34^ exact stimulation period,^35^ the structure of the brain area,^29^ type of cognitive task^36^ and stimulation intensity,^37^ among other parameters. Recent studies have also highlighted the importance of history-dependent and state-dependent effects.^38^ For example, the effect of stimulation also depends on the initial state of the area being stimulated,^39^ number of previous pulses delivered^39^ and the specific neural subpopulations that are recruited by the behavioral task that accompanies the TMS.^40^

In summary, non-invasive brain stimulation techniques, including TMS and tES, provide a powerful approach for linking brain to behavior: by perturbing brain activity and measuring its effects on behavior. However, there are essential gaps in knowledge regarding the mechanisms by which these techniques affect brain activity and, consequently, behavior. Nevertheless, recent technological advances and combinatorial paradigms (reviewed in Sects. 5, 6) are fast closing these knowledge gaps and neurostimulation is emerging as an indispensable tool for understanding the causal mechanisms underlying cognitive phenomena, including selective attention, in humans.

## Control of Attention: Top-Down and Bottom-Up

Top-down attention is under voluntary control, and allocated according to internal behavioral goals and, hence, is also known as “goal-directed” attention. Conversely, bottom-up attention is automatically captured by salient stimuli, typically overriding internal goals and, hence, is also known as “reflexive” attention.^41^ Top-down visuospatial attention takes longer to deploy (~300 ms) and can be sustained for as long as the task demands. On the other hand, bottom-up attention is more rapid, but also more transient—rising by 120 ms and then falling off typically within 300 ms.^42^ This faster neural time course and reflexive nature have led to the hypothesis that bottom-up attention is mediated by a distinct, phylogenetically older attention system that allows organisms to quickly orient and respond to salient, novel or dangerous stimuli.^43^

Are top-down and bottom-up attention indeed mediated by distinct brain regions and mechanisms, or by a common region which dynamically modulates activity and connectivity according to task demands? In this section, we first review evidence from human psychophysics studies showing key similarities and differences between the behavioral effects of top-down and bottom-up attention. Next, we review evidence from neuroimaging and electrophysiology studies that investigate similarities and differences in the neural substrates of the two modes.

### Behavioral Effects of Top-Down and Bottom-Up Attention

The Posner cueing task is among the earliest, and most widely applied, psychophysical paradigms for exploring differences between top-down and bottom-up attention. The Posner paradigm employs either a central or peripheral cue that precedes a target stimulus, which subjects have to detect. Central cues indicate the most likely location of the upcoming target and engage top-down attention towards the cued side. On the other hand, a transient, peripheral cue, like a brief (e.g., 50 ms) flash, at one of the possible locations of the forthcoming target automatically engages bottom-up attention at that location, even if the cue is not predictive about the subsequent target. The effects of cueing are measured in terms of changes in behavioral accuracy and reaction times at the cued versus uncued locations. The Posner paradigm, thus, allows studying the behavioral consequences of both engagement and disengagement of attention. Moreover, both of these conditions can be compared against a baseline established by a neutrally cued version of the task.^44^ With this paradigm, previous studies have investigated the effects of attention on various aspects of perception including contrast sensitivity, orientation sensitivity, spatial resolution, texture segmentation, temporal resolution and the like, thereby providing insight into their mechanistic underpinnings.^42^^,^ 45 ^–^ 50

**Figure 1:**
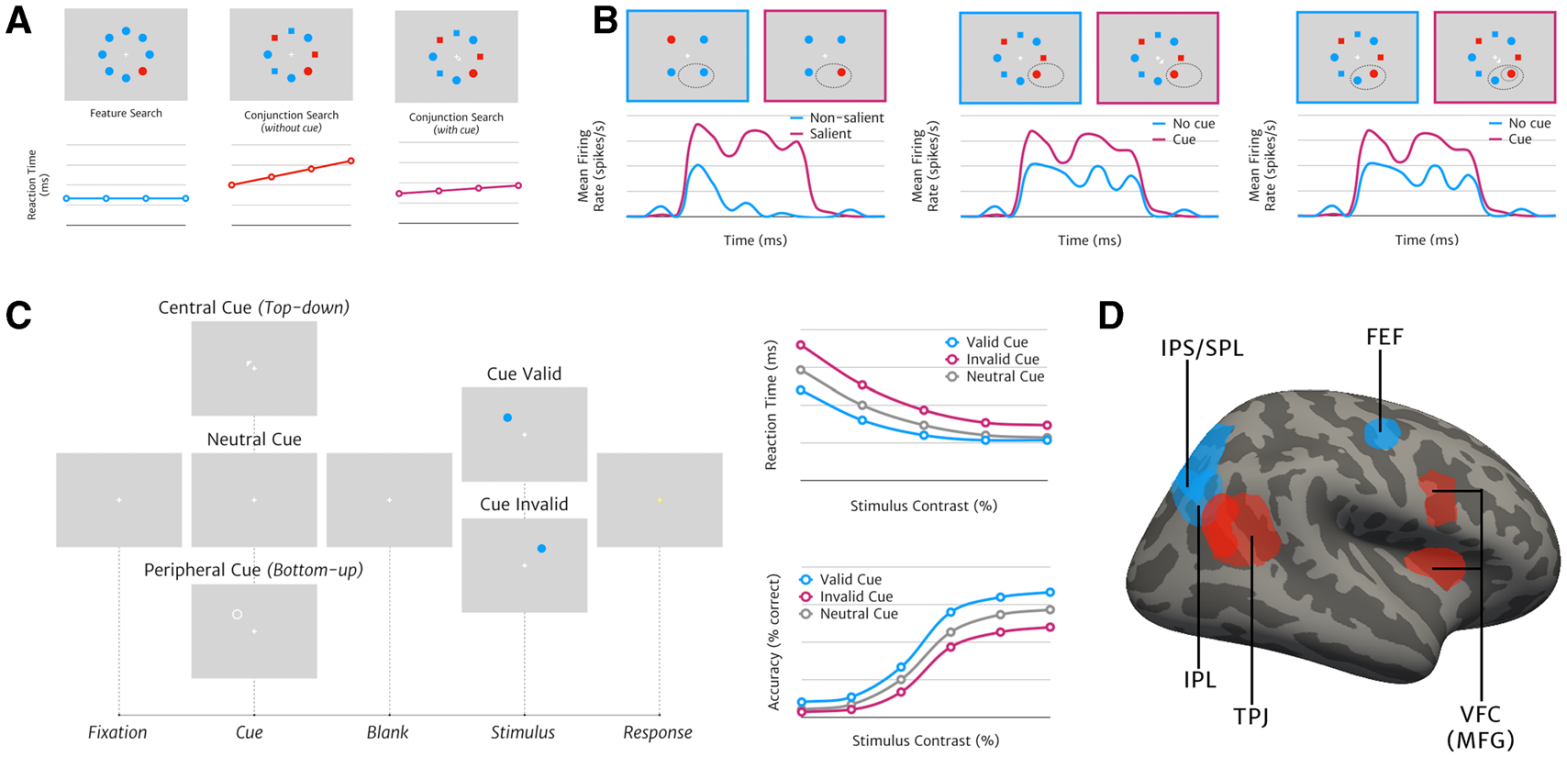
Attention’s effects on brain and behavior. **a** (Left) Pop-out (bottom-up) search task. Target differs from distractors in a single salient feature (color singleton); (below) Reaction time (RT) does not increase with number of distractors (set size). (Middle) Conjunction (top-down) search task without cueing. Target differs from distractors based on a conjunction of features (color and shape); (below) RT increases with set size. (Right) Conjunction search task with central (top-down) cue indicating location of target; (below) RT increases marginally with set size. **b** (Left) Schematic of neuronal firing in visual and attentional areas when the neuron’s receptive field (RF, dashed black oval, upper panel) contains a non-salient stimulus (lower left panel and blue trace) versus a salient stimulus (lower right panel and purple trace). (Middle) Same as in the left panel, but when a top-down cue is used to direct attention to a stimulus within its RF (lower left panel and blue trace) versus outside the RF (lower right panel and purple trace). (Right) Same as in the left panel, but when a distractor is present along with the target in the neuron’s RF. The suppression of activity caused by the distractor (lower left panel and blue trace) can be alleviated by directing attention specifically to the target (lower right panel and purple trace). **c** (Left) Posner cueing paradigm. Fixation is followed by the appearance of a cue. The cue can be a central or top-down cue (arrowhead, upper panel), a neutral cue (middle panel) or a peripheral or bottom-up cue (transient flash, lower panel). This is followed by the appearance of the stimulus, after a brief delay. Subjects have to detect the presence, identify or localize the target stimulus, which may appear on the cued side (validly cued trials) or not (invalidly cued trials). (Right, upper) Reaction times typically decrease with increasing target strength (e.g., stimulus contrast). The reaction times are highest for invalidly cued trials, intermediate for neutrally cued trials, and least for validly cued trials. (Right, lower) Accuracy (% correct) is typically least for invalidly cued trials, intermediate for neutral cues and highest for validly cued trials. **d** Important nodes in frontal and parietal cortex involved in attention. Areas in blue are primarily involved in top-down control of attention, but also activate, albeit less strongly, during bottom-up attention. Areas in red are primarily implicated in bottom-up, stimulus-driven reorienting (abbreviations expanded in main text).

Top-down and bottom-up attention have several similar behavioral consequences. First, both top-down and bottom-up attention produce a benefit, in terms of higher accuracies and shorter reaction times (RT), for detecting targets at the attended (cued) location, as compared to neutral conditions^43^ (Fig. 1c). Both types of attention also induce a corresponding cost (lower accuracy, higher RT) at the unattended side compared to the attended side,^51^^–^ 54 indicating that both modes of attention involve selective allocation of limited cognitive resources. This common underlying mechanism is thought to operate by biasing competition for neural resources in favor of the attended stimulus/location.^55^^,^ 56 Second, both top-down and bottom-up attention increase contrast sensitivity to target stimuli when presented alone or concomitantly with distractors. Moreover, both types of attention have been reported to enhance the perceived contrast of the attended stimulus,^43^^,^ 53, suggesting that both mechanisms influence visual processing of target stimuli. Finally, both top-down and bottom-up attention increase spatial resolution at the location of target stimuli to facilitate their discrimination, for example, in tasks that demand high visual acuity judgments.^42^^,^ 53 ^,^ 57 ^–^ 59

Despite these similarities, several key differences have been reported between these attention modes.

First, differences are observed in terms of the inhibition-of-return (IOR) effect, which describes the tendency for attention to not be re-deployed to a location or stimulus that was recently selected: targets that immediately follow the presentation of a bottom-up cue (< 150 ms) are more readily detected compared to those appearing after a significant delay (> 300 ms).^52^ Nevertheless, recent evidence suggests that IOR can also occur under top-down control. For instance, in detection tasks, IOR occurs only for frequent targets, but not for infrequent (odd-ball) ones, indicating the involvement of top-down processes in modulating IOR.^60^

Second, the two modes of attention operate differently with predictive validity of the cue, i.e., the probability of the target appearing at the cued location. In top-down attention experiments, the preferential allocation of attention to a location depends upon cue validity: a location with higher validity is afforded higher priority, and attentional benefits on performance measures (e.g., accuracy and RT) in top-down tasks systematically vary with cue validity. In bottom-up tasks, as cues are usually spatially non-predictive and attention is automatically captured, performance is typically similar across locations. However, surprisingly, even when bottom-up cues are spatially predictive of target appearance benefits and costs in terms of accuracy and reaction times largely remain similar across cue validities, suggesting that the faster timescale bottom-up effects operate independently of slower top-down effects.^58^^,^ 61

Third, early studies reported differences in terms of their respective effects on the psychophysical function: top-down attention was shown to operate via contrast gain (a shift in contrast threshold), whereas bottom-up attention was shown to operate via response gain (a shift in performance asymptote).^42^ However, more recent studies have employed the normalization framework to challenge these results^62^: both types of attention can engage either contrast gain or response gain mechanisms depending on the size of the attention field relative to the size of the target stimulus.^51^

Finally, whereas bottom-up attention exclusively increases spatial resolution at the attended location, top-down attention adaptively alters (increase or decrease) spatial resolution depending on task demand. For instance, in a texture segmentation task that required integration of information from an extended area around the attentional focus, bottom-up cues enhanced spatial resolution at the focus and thereby hindered performance by limiting the spatial integration window around the focus. On the other hand, top-down cues permitted adaptively increasing or reducing the spatial integration window by modulating spatial resolution as was optimal for the task.^50^^,^ 58 In addition, bottom-up attention reduces temporal resolution even while increasing spatial resolution, thereby compromising fine temporal judgments.^63^ These results have led to the idea that top-down attention is a more flexible and adaptive system than bottom-up attention.

In addition to Posner cueing, visual search paradigms have also been used for studying the psychophysics of top-down and bottom-up attention. Visual search typically involves finding a known target stimulus among irrelevant distractors in a cluttered display. If the target’s features are widely different from distractors’ features, the high visual salience of the target captures attention through a bottom-up mechanism—a phenomenon termed “pop-out”. On the other hand, when targets and distractors share many featural similarities (“conjunctions”), identifying the target requires the active deployment of top-down attention (Fig. 1a).

Bottom-up and top-down attention mechanisms, engaged by pop-out and conjunction search, respectively, produce distinct behavioral consequences. In pop-out search, increasing the number of distractors typically has no effect on the time taken to find the target, whereas in conjunction search the time to find the target increases with the number of distractors. Bottom-up attention can also impair behavioral performance in search tasks by counteracting the effects of top-down attention. In a task involving detecting a target based on its unique shape (a shape singleton) the presence of color singletons diverted attention in a bottom-up manner; thus bottom-up distractors reduce detection efficiency in top-down search tasks. Nevertheless, top-down attention is able to overcome the distracting effect of bottom-up distractors, typically within around 200 ms.^64^^,^ 65

Differences also exist between top-down and bottom-up attention in terms of their relative benefits for conjunction versus feature search. It has been found that bottom-up attention recruited through peripheral cues causes greater effects on conjunction searches than on feature searches, while this difference is not seen for central cues, during top-down attention. On the other hand, the meridian crossing effect—a higher behavioral cost when the cue and target are proximal, but on opposite sides of the vertical meridian—is seen for top-down, but not for bottom-up attention.^61^

Taken together, these studies indicate that top-down and bottom-up attention produce overlapping, but not identical behavioral effects. Identifying the distinct neural correlates of top-down and bottom-up attention is the logical next step toward teasing apart specific mechanisms of the two modes of attention, and this is discussed next.

### Neural Correlates of Top-Down and Bottom-Up Attention

A variety of brain regions are thought to be actively involved in visuospatial attention. These include cortical regions such as the prefrontal, and parietal cortex^41^ as well as sub-cortical regions, including the superior colliculus,^66^ thalamus,^67^ basal ganglia^68^ and mesolimbic structures.^69^ These regions are hypothesized to mediate attention’s effects by altering the coding of “selected” neural information in sensory^70^^,^ 71 and decision areas.^72^ However, whether these regions play distinct, separable roles in top-down versus bottom-up attention remains debated. Reports range from highly overlapping neural substrates,^73^ to a near-complete dissociation.^74^ We review here brain imaging (fMRI) and lesion studies in human subjects as well as electrophysiology studies in non-human primates that investigate the neural bases of the two modes of attention.

There is clear evidence from lesion studies for the involvement of the prefrontal cortex and parietal cortex in both top-down and bottom-up attention (Fig. 1d). Patients with unilateral or bilateral lesions in the frontal eye fields (FEF), dorsolateral prefrontal cortex (DLPFC) and posterior parietal cortex (PPC) exhibit ‘spatial neglect’ syndromes, in which patients are unable to attend to and, hence, detect contralesional visual stimuli. The deficits can be ameliorated by both top-down and bottom-up cues, for instance, by instructing patients verbally to attend to stimuli in the area of neglect, or by presenting salient stimuli, such as noises in the affected area.^41^

Functional MRI studies have also shown that fronto-parietal regions, including the FEF and DLPFC, the intraparietal sulcus (IPS), and temporoparietal junction (TPJ) are active during both top-down and bottom-up attention tasks. In top-down attention tasks, these regions show robust activation especially in the anticipatory period after the cue and before target appearance. Moreover, the intensity of activation in these areas increases as top-down cues became more predictive.^74^ The same areas show activation during bottom-up attention also, although the strength of activation was weaker when compared to the top-down attention case.^75^ Reinforcing these results, Peelen et al employed a task involving detection of a target in a Posner-like cueing paradigm with concurrent fMRI. They tested subjects using both central (top-down) and peripheral (bottom-up) cues and showed that a single holistic network controls both top-down and bottom-up orienting of attention. This network included the fronto-parietal regions mentioned above, as well as the right inferior frontal gyrus (IFG), anterior cingulate cortex (ACC), premotor cortex, bilateral precuneus and cerebellum. Comparing activity levels in these network regions yielded no significant differences between the two modes of orienting, except for the TPJ, which showed a slightly higher activation upon bottom-up cueing.^73^

In contrast to these findings, a study by Hahn et al.^74^ showed that distinct brain networks underlie top-down and bottom-up attention. The ‘spatial attention resource allocation task’ in this study employed varying degrees of validity of a central cue to selectively recruit top-down (high cue validity) and bottom-up (low cue validity) attention, which enabled them to test a range of activation levels for both modes. Specifically, they showed that left and right middle frontal gyrus, left inferior and superior parietal lobule (IPL, SPL; near the IPS) and bilateral precuneus were engaged by the top-down attention task, whereas areas TPJ, right anterior and posterior insula, left and right fusiform gyrus and anterior cingulate gyrus were engaged by the bottom-up attention task. The authors attributed the differences between their results and those of previous studies to differences in task design, and suggested that tasks that could not adequately distinguish activity evoked by the cue from that evoked by the target tended to report overlapping or common networks for both attentional modes.^74^

Electrophysiological studies have also contributed significantly to understanding similarities and differences in the neural mechanisms of top-down and bottom-up attention. In general, both modes of attention enhance the neural encoding of target stimuli, and suppress the encoding of distractors.^65^ These effects can occur through enhancing firing rates of neurons, altering inter-neuronal firing rate correlations within a local neural population or generating synchronized activity across distal neural populations.^70^^,^ 76 Whereas human fMRI studies have investigated the involvement of distinct brain regions in top-down versus bottom-up attention, studies based on monkey electrophysiology have primarily shed light on the distinct dynamics of the two modes of attention. For instance, in macaque area MT, neural modulation induced by bottom-up attention has a faster time course than that induced by top-down attention.^77^

The fronto-parietal cortex shows clear electrophysiological signatures of both bottom-up and top-down attentional selection (Fig. 1b). In the case of bottom-up attention, when a salient stimulus stands out from surrounding stimuli (e.g., pop-out) it captures bottom-up attention, and is encoded more strongly, in terms of greater spike rates, across all levels of visual processing, from V1 to the PFC.^78^ In the case of top-down attention, when multiple stimuli are presented within the receptive field top-down attention biases the competition towards encoding the goal relevant stimulus; fronto-parietal areas are thought to mediate this attentional biasing.^55^ Moreover, during bottom-up (pop-out) and top-down (serial) search, attentional enhancement of neural activity in FEF precedes attentional enhancement in visual cortex, indicating selection in FEF occurs earlier than in the visual cortex.^61^^,^ 70 Finally, microstimulation of the FEF or the LIP causes attention like effects in the firing rates of visual cortex neurons, indicating a common mechanism for the action of both attention modes.^79^^,^ 80

A primary difference between bottom-up and top-down attention in the fronto-parietal cortex appears to be in the relative timing of neural activation for discrimination of the selected target. Recording from the lateral intraparietal area (LIP) and the frontal eye fields (FEF), Buschmann and Miller showed that neural signatures of selection emerged earlier in the LIP compared to FEF during bottom-up attention, whereas the reverse order of activation was observed during top-down attention.^76^ Similarly Ibos et al. found that as the level of top-down information required in an orientation detection task increased, faster responses were observed in FEF neurons than LIP neurons.^78^^,^ 81 ^,^ 82 Confirming these trends, human EEG studies have shown that during top-down attention, frontal cortical signals preceded specific parietal cortical signals. Grent-t’-Jong et al. used fMRI seeded ERP/EEG source localization models to show that frontal contribution to an orienting-specific electrical wave of activity began by 400 ms post-cue while that of the parietal cortex contribution occurred only at 700 ms.^83^ However, other studies have found no difference in neural activation times between the frontal and parietal cortices^84^^,^ 85 and these temporal order effects in top-down versus bottom-up attention remain debated.

Attention also exerts systematic effects on neural oscillations, although these investigations have been primarily confined to top-down attention studies. Ikkai et al. 2016, recorded MEG in the occipital cortex as the subjects performed a discrimination task, attending covertly using a cue that either predicted the target location with absolute certainty or did not provide any information. Alpha desynchronisation was observed contralateral to the attended location when the cue was predictive, but bilaterally when the cue was neutral, suggesting a top-down control of attention over visual encoding mediated by alpha oscillations.^86^ Similar effects in the degree of laterality of alpha desynchronisation were observed using EEG.^87^^,^ 88 In addition, studies showing increase in alpha power ipsilateral to the attended location,^89^ have also proposed that alpha oscillations work as a gating mechanism for selective processing of information.^89^ Similarly, increase in gamma power in the visual cortex has been observed as an effect of top-down attention, both with EEG and MEG.^90^^,^ 91

A few EEG/MEG

**EEG/MEG:** Electro/Magnetoencephalography—non-invasive techniques for recording, with high temporal resolution, changes in electric and magnetic fields at the level of the scalp induced by the concerted activity of large neural populations.

and local field potential (LFP) studies have also sought to identify distinct signatures of top-down and bottom-up attention. For instance, Landau et al.^92^ reported that only top-down, voluntary shifts of attention, but not bottom-up shifts, increased gamma power in the contralateral fronto-parietal regions, supporting different mechanisms of action of the two modes of attention.^92^ In contrast, the study by Buschmann and Miller^76^ showed that bottom-up attention (pop-out search) induced synchronization in a high-frequency gamma band (35–55 Hz) between frontal and parietal areas, whereas top-down attention (serial search) induced synchronization at lower frequencies (22–34 Hz).^76^ The authors suggest that bottom-up selection could be mediated by high-frequency communication among proximal brain areas, whereas top-down attention may be mediated by lower frequency communication among more distal areas.

In summary, there is emerging consensus that top-down and bottom-up attention are mediated by at least partially distinct neural substrates. Even when neural substrates are common across the two modes, there is emerging evidence for key differences in time courses of neural activation within these regions. The spatial and temporal resolution of TMS provides a unique opportunity to test the causal involvement of these different brain regions, differences in the precise timing of their activation, as well as characteristic oscillatory signatures during top-down and bottom-up attention.

## Top-Down Versus Bottom-Up Attention Mechanisms Investigated with TMS

### Mechanisms of Top-Down Attention

The vast majority of TMS experiments have investigated the neural basis of top-down attention. Specifically, many studies have investigated the role of the fronto-parietal cortex by applying rTMS to perturb activity in the FEF and/or PPC during perceptual detection, discrimination or attention tasks and analyzed the effects on behavioral performance.

Grosbras and Paus applied single-pulse TMS over FEF shortly before target onset in a visual detection task and found that it increased detection rates by increasing visual sensitivity, conferring the ability to detect previously undetected visual stimuli.^93^ Effects were seen only for contralateral stimuli for left FEF stimulation, but bilaterally for right FEF stimulation. Single-pulse TMS on the FEF just before target onset during a detection task also facilitated visual awareness of targets and reduced the time taken to detect them.^94^ Neggers et al. applied three TMS pulses (30 ms intervals) to the right FEF 30 ms before target presentation; their task tested the ability of subjects to discriminate targets towards which they were already preparing a saccade. Here, TMS led to decreased discrimination accuracy on the contralateral side, perhaps by disrupting saccade preparation towards contralateral stimuli, and thereby disrupting attention toward that side.^95^ These conflicting findings may reflect different effects of single-pulse versus triple-pulse TMS.

During a centrally cued (top-down) target detection task, single-pulse TMS applied 53 ms before the target onset to the left or the right FEF led to a shortening of reaction times. Left FEF stimulation produced RT effects only for contralaterally presented stimuli and regardless of cue condition (valid/neutral/invalid), whereas right FEF stimulation produced RT effects for both contralateral and ipsilateral stimuli, but only for validly or neutrally cued conditions. This performance enhancement effect was interpreted as an enhancement of cueing benefits on the valid side, indirectly leading to an increase in cost of invalid cueing.^94^ On the other hand, applying 5 pulses of TMS at 20 Hz over the left FEF 50 ms prior to cue onset in a centrally cued detection task took away the cost of invalid cueing: performance on invalidly cued sides improved, although performance on the validly cued side was unchanged.^35^ These different results have been reconciled as follows: when cueing benefit effects reach a ceiling, cueing costs may be alleviated by the FEF through attention.

Studies have also shown the involvement of other frontal areas (such as DLPFC and the medial frontal cortex) in top-down processes like switching attention between different tasks, working memory and change detection. For example, in a study where subjects had to report changes between two images separated by a 300 ms blank, an rTMS train of eight pulses at 10 Hz over the right DLPFC reduced accuracy for detecting changes.^96^ Kalla et al. also demonstrated the involvement of the DLPFC in conjunction, but not feature searches; they observed decreased conjunction search accuracy following cTBS suppression of the DLPFC.^97^ Rushworth et al. further tested the involvement of the pre-supplementary motor area in task switching, in which subjects were cued to follow one of two stimulus–response rule sets. A four-pulse rTMS train at 5 Hz applied over the pre-supplementary motor area following the cue impaired task performance only in conditions where the subject had to switch to a different stimulus–response rule. This indicates prefrontal causal involvement in top-down stimulus response mapping.^25^ Similarly, Muggleton et al. have also shown increased response times in switch tasks during rTMS of the FEF (5 pulses at 10 Hz).^98^ These studies indicate a role of frontal regions in controlling many different top-down cognitive processes associated with attention and visuospatial processing.

Similar studies have been conducted on the PPC, but with more disruptive than facilitative outcomes. Fugetta et al. applied single-pulse TMS on the right posterior parietal cortex (rPPC) after target onset during a conjunction search task and found that it delayed response times to targets.^99^ Thut et al. in 2005, used an offline protocol involving continuous rTMS at 1 Hz for 25 min on the right PPC, with the aim of suppressing activity in this region, following which subjects performed a visual target localization task involving top-down cues. Following rTMS subjects exhibited impaired detection for all leftward cued trials, both valid and invalid, while rightward cued trials showed enhanced valid and impaired invalid target detection, indicating a role of right PPC in voluntary leftward orienting (leftward validly cued stimuli), and global reorienting (all invalidly cued stimuli).^100^ Similarly, Beck et al. applied 500 ms trains of 10 Hz rTMS pulses over the right and left PPC, while subjects tried to detect changes between two images separated by a 100 ms blank. They found that right PPC rTMS caused longer change detection latencies (increased RTs) as well as lowered detection rates.^101^ Several other studies have used concurrent TMS-fMRI/ERP to investigate the precise roles of both PPC and FEF; these discussed in Sect. 5.

**Figure 2:**
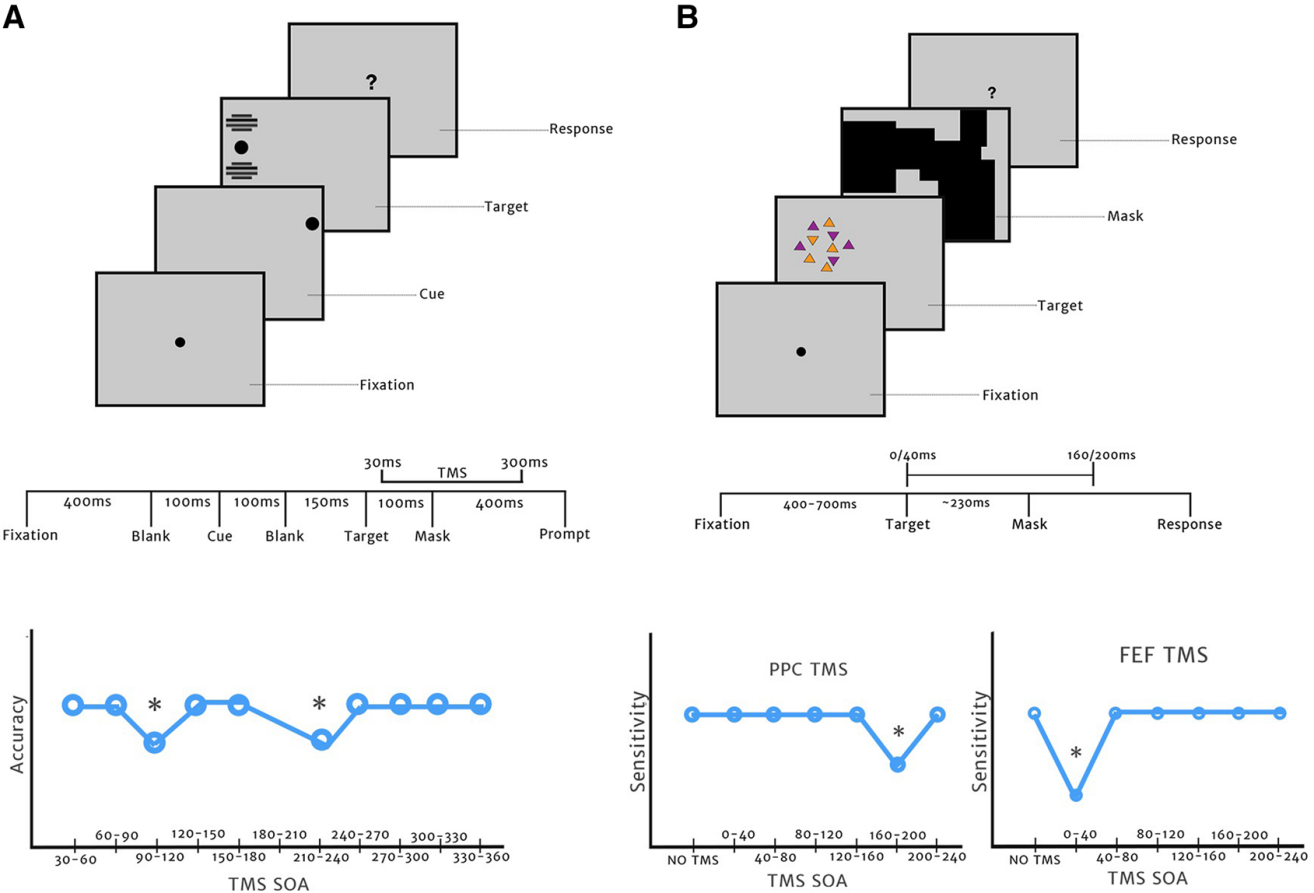
TMS effects on behavioral performance in top-down and bottom-up paradigms. **a** Effect of single-pulse TMS on the PPC in a bottom-up localization task. (Chambers et al.^24^). (Top) Task structure: following fixation, a brief peripheral cue (50 ms) appeared. After this, a cue-like stimulus reappeared along with grating stimuli above and below it, either on the same side as the cue (validly cued trials) or side opposite to the cue (invalidly cued trials). Subjects were required to indicate the vertical position of the grating with the higher frequency. (Middle) Timeline of a representative trial. A single pulse of TMS was delivered to the PPC at one of 12 different stimulus-onset asynchronies (SOAs), ranging from 30 to 360 ms, following target onset. (Bottom) Performance (percent correct) during invalidly cued trials as a function of TMS delivery SOA. Performance dropped significantly for 90–120 and 210–240 ms SOAs indicating specific critical timings of parietal cortex involvement in orienting bottom-up attention. No TMS effects on performance were observed for valid trials. **b** Effect of double-pulse TMS on the PPC and FEF in a conjunction search (top-down) task (Kalla et al.^104^). (Top) Task structure. After a variable fixation period, from 400 to 700 ms, a search array was presented. The search array consisted of ten elements. After a brief period of search array presentation (average duration ~220 ms; titrated for individual subjects) a visual mask was presented. Subjects had to report the presence or absence of the target by key press. (Middle) Timeline of a representative trial. Double-pulse TMS was delivered over PPC or FEF at one of 5 pairs of SOAs after search array onset (0/40, 40/80, 80/120, 120/160, 160/200 ms). (Bottom) Target detection performance (represented by perceptual sensitivity in the y axis) as a function of TMS delivery SOA (pairs). Performance decreased significantly for 0/40 ms SOA TMS upon FEF stimulation and at 120/160 ms upon PPC stimulation compared to no TMS condition.

TMS has also been used with visual search and pattern recognition paradigms to understand the time course of top-down attention in the fronto-parietal cortex. Single-pulse TMS was applied to disrupt activity in the posterior part of the inferior parietal lobule during a conjunction search task.^102^ Stimulation caused a significant delay in target identification times when applied 100 ms from the onset of the search array, but not at other onset asynchronies (0–200 ms), indicating the precise time course of PPC involvement in top-down search. On the other hand, double-pulse TMS applied at the FEF during a conjunction search showed inhibitory effects as early as 40 ms post search array onset, suggesting an earlier involvement of the FEF compared to the PPC in top-down visual search.^103^ Kalla et al. have also used double-pulse TMS on the FEF and PPC and seen similar effects^104^ (Fig. 2b). Similarly, double-pulse TMS with an interstimulus interval of 100 ms was applied over the left and right PPC during a visual pattern recognition task at a range of target onset asynchronies (120–520 ms). Inhibitory effects on performance were found for right PPC stimulation applied 270 ms post target onset. Since the task involved complex cognitive components, such as object recognition and response mapping, the authors inferred that disrupting late PPC attentional mechanisms were responsible for these results.^105^ Overall, the results support an earlier engagement of FEF compared to PPC in top-down attention tasks.

### Mechanisms of Bottom-up Attention

Studies investigating bottom-up control of attention with TMS fall into one of three categories: those investigating bottom-up cueing of attention, stimulus competition or stimulus-driven reorienting of attention.

Very few studies have directly investigated neural mechanisms of bottom-up cueing of attention. A notable example is the study by Chambers et al. who stimulated the angular gyrus, situated in the inferior parietal lobule (IPL), with single-pulse TMS during a target localization task with bottom-up cueing (Fig. 2a). Subjects’ accuracies (percent correct) in invalidly cued trials were affected at two stimulation time periods relative to target onset: one early (90–120 ms) and one late (210–240 ms). The results were ascribed to TMS-induced disruption of bottom-up processing in two feed-forward visual information streams, an early one from the superior colliculus and a relatively late one from the striate pathways to the PPC.^106^

The involvement of the PPC in bottom-up stimulus competition, operating across hemifields, has been more extensively studied. Stimulation of the TPJ has been used to mimic symptoms of hemi-extinction, in which patients with parietal cortex lesions tend to ignore stimuli in the contralesional visual hemifield, especially when stimuli are presented concurrently in the ipsilesional hemifield; a phenomenon thought to be mediated by bottom-up stimulus competition across visual hemifields. A study by Meister et al. applied single-pulse TMS to the right TPJ and superior temporal gyrus (STG), while subjects attempted to localize dot stimuli that appeared either unilaterally or bilaterally. TMS introduced extinction like effects in the left hemifield during bilateral stimulus presentation only when applied to the right TPJ but not when applied to right STG.^36^ Single-pulse TMS was used to stimulate left and right parietal cortex both unilaterally and bilaterally (concurrently) by Dambeck et al. during localization of dot stimuli. Unilateral, but not bilateral, rTMS caused deficits in detection of contralateral stimuli, suggesting that stimulation of both hemispheres offset the relative effects of suppression in each other and led to no net behavioral change.^107^ Using a similar protocol, Hilgetag et al. applied rTMS at 1 Hz for 10 min over the right and left PPC, while subjects had to detect the presence of small squares presented either bilaterally or unilaterally. For right PPC stimulation, they found not only an overall decrease of correct responses for bilaterally presented stimuli, but also increased detection performance for ipsilateral stimuli, with concurrent decreased performance for contralateral stimuli, during unilateral presentations.^108^ In this study, right PPC stimulation showed a much larger effect compared to left PPC stimulation, suggesting that the right PPC may have a stronger involvement in bottom-up visuospatial processing.

**Figure 3:**
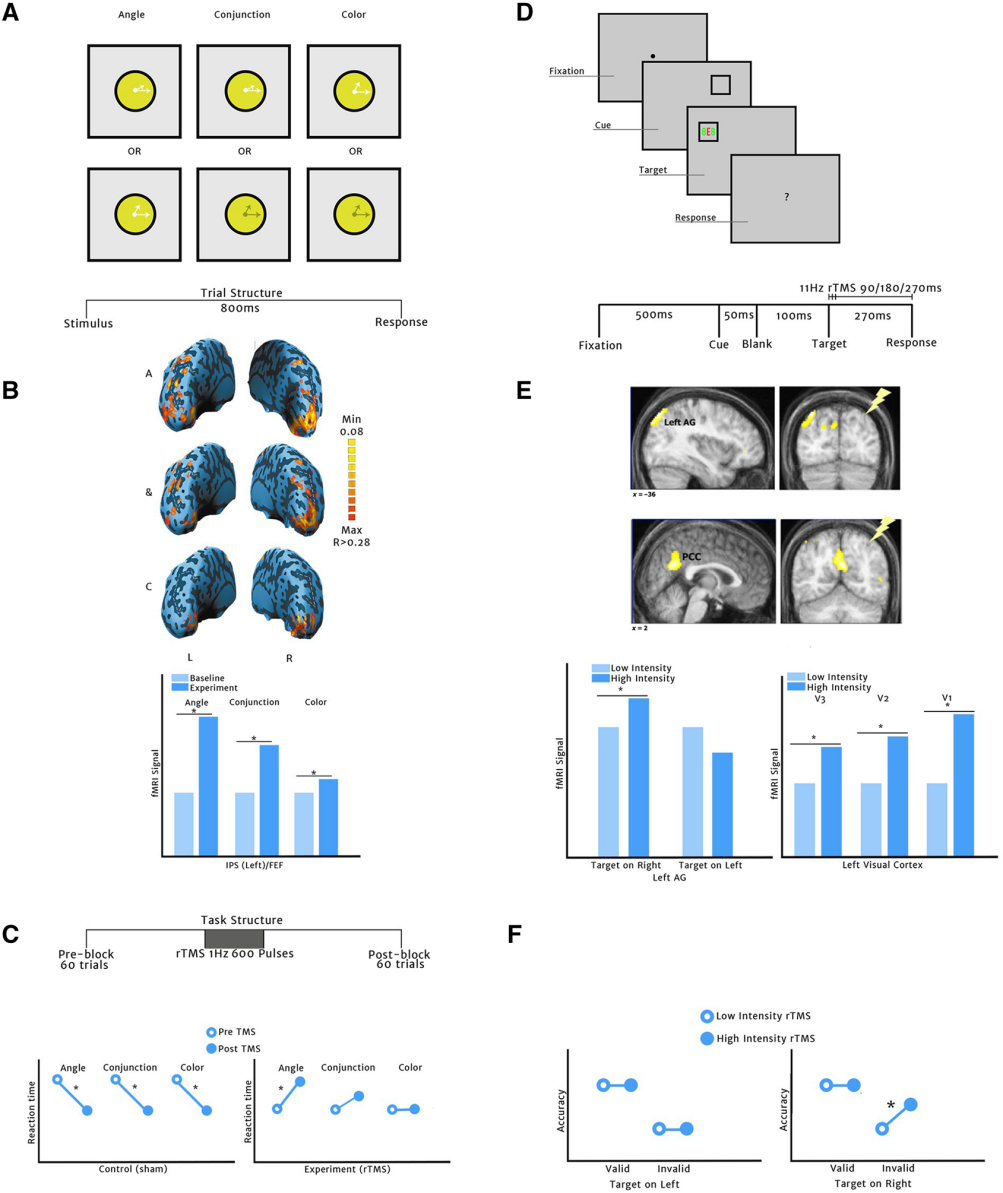
TMS effects on neural signals (fMRI) and behavioral performance in top-down and bottom-up paradigms. **a** Effects of rTMS over PPC on behavior and fMRI activation during top-down discrimination (Sack et al.^139^). (Top) Task structure: clock stimuli were presented with the hands in a specific color and/or subtending a specific angle. Subjects had to identify the presence or absence of a particular subtended angle (e.g., 60°), in an angle discrimination task, or a particular color of the hands (e.g., white), in a color discrimination task, or a combination of both (conjunction discrimination task). Each type of task occurred in a continuous block structure. (Bottom) Timeline of a representative trial. Each trial involved 800 ms of stimulus presentation followed by a response. **b** (Top) fMRI BOLD activation maps during angle discrimination (upper), conjunction (middle) and color discrimination task performance (lower). (Bottom) fMRI BOLD signal in the parietal cortex (left and right IPS) increased during the angle and conjunction discrimination tasks much more than for the color task (relative to no task baseline). **c** (Top) Representative timeline of the experiment. Following a control (pre-TMS) behavioral session, rTMS or sham stimulation was delivered at 1 Hz (600 pulses) over the PPC, immediately followed by a post-TMS behavioral session. (Bottom) Effects of rTMS on task performance. (Left) Following sham TMS RT decreased for all three task types, due to practice effects. (Right) Following rTMS this decrease in RT was obliterated for angle and conjunction discrimination tasks, with reaction times increasing significantly for the angle discrimination task. **d** Effects of right angular gyrus (AG) rTMS on BOLD signals and behavior during a bottom-up letter discrimination task (Heinen et al.^109^). (Top) Task structure. Following fixation, a brief (50 ms) bottom-up cue (box) was presented. The target, consisting of a red or green letter (E/A/F/P) flanked by similar red/green shapes, appeared after a delay of 100 ms. Subjects had to indicate the identity of the letter within a 3 s response interval. (Bottom) Timeline of a representative trial. rTMS (3 pulses at 11 Hz), either at low or high intensity, was delivered at one of three SOAs (90, 180 or 270 ms) after target onset. **e** (Top) fMRI BOLD activation maps during task performance post high intensity rTMS of right AG. (Bottom) (Left) BOLD signals in the left AG increased for right targets, and decreased for left targets following right AG rTMS. (Right) BOLD signals increased in left V1, V2 and V3 post high intensity rTMS, compared to low intensity rTMS of right AG. **f** Effect of rTMS on accuracy during validly and invalidly cued trials. The main effect was an increase in accuracy for invalidly cued targets on the right, following high intensity rTMS of the right AG.

Finally, several studies have addressed the role of the ventral PPC, including the IPL and TPJ, in bottom-up, “stimulus-driven reorienting”. In both top-down and bottom-up attention tasks (e.g., Posner cueing tasks), the target stimulus can occur at a location different from the cued (attended) location; such trials are termed invalidly cued trials (Sect. 3.1). In these trials attention must be quickly reoriented from the cued location toward the uncued location at which the target occurred, a phenomenon termed stimulus-driven reorienting. TMS over the right PPC has been shown to consistently affect such bottom-up reorienting. For instance, in a target discrimination task in which subjects had to identify a target letter appearing either on the left or the right, preceded by non-predictive bottom-up cues, a burst of three TMS pulses at 11 Hz delivered over the right angular gyrus (AG) 90–270 ms following target onset improved performance specifically at invalidly cued target locations on the right hemifield.^109^ Concurrent fMRI performed during the same study showed activation of the left AG following right AG rTMS, suggesting that stimulus-driven reorienting may be mediated by cross-hemispheric competition in the parietal cortex (Fig. 3d–f). Similarly, in a top-down cued, letter discrimination task, 150 ms of rTMS (20 Hz) delivered over the right IPS during the cue period, putatively to suppress activity in the IPS, produced deficits in identification performance mostly when the target appeared at invalidly cued locations.^110^ This effect, unlike in the AG, was seen for invalidly cued targets in both right and left visual hemifields. Moreover, the TPJ, has been implicated in attentional reorienting under both top-down and bottom-up conditions.^41^ cTBS applied over the right anterior TPJ impaired reorienting in an endogenously cued visual task^111^.

In addition, the parietal cortex has also been implicated in top-down modulation of bottom-up processes. TMS applied over the visual cortex can elicit the perception of brief flashes or phosphenes, which is thought to arise from bottom-up (stimulation-driven) activation of the visual cortex neurons; the minimal strength of stimulation required to elicit phosphenes ~50% of the time is referred to as a phosphene threshold. The phosphene threshold at specific spatial locations is known to be reduced when top-down attention is directed to those locations.^112^ Silvanto et al. used a dual stimulation paradigm with two TMS coils: one to stimulate the angular gyrus and one to concurrently stimulate the early visual cortex (V1/V2). They found that triple pulse stimulation of the angular guys lowered the threshold for TMS-evoked phosphenes in the early visual cortex, in a manner analogous to top-down attention’s effect on phosphene thresholds.^113^ These results indicate that the angular gyrus may recruit top-down control mechanisms that modulate bottom-up activation of the visual cortex.

### Role of Neural Oscillations in Attention

In addition to probing the causal role of specific brain regions, the temporal precision of TMS has been exploited to investigate the role of fast brain oscillations that accompany attentional states. Attention is known to suppress the power of alpha-band (8–12 Hz) oscillations in the brain hemisphere contralateral to the attended location^114^; attention also enhances gamma-band (30–90 Hz) oscillations in the hemisphere contralateral to the attended location^115^ (see Sect. 3.2 for more details). As the vast majority of these studies have been conducted with top-down cueing we do not explore further the dichotomy between oscillatory correlates of top-down and bottom-up attention in this section.

Both online and offline TMS protocols have been used to study the causal involvement of alpha and gamma oscillations in attention. These can be broadly grouped into two categories.

**Figure 4:**
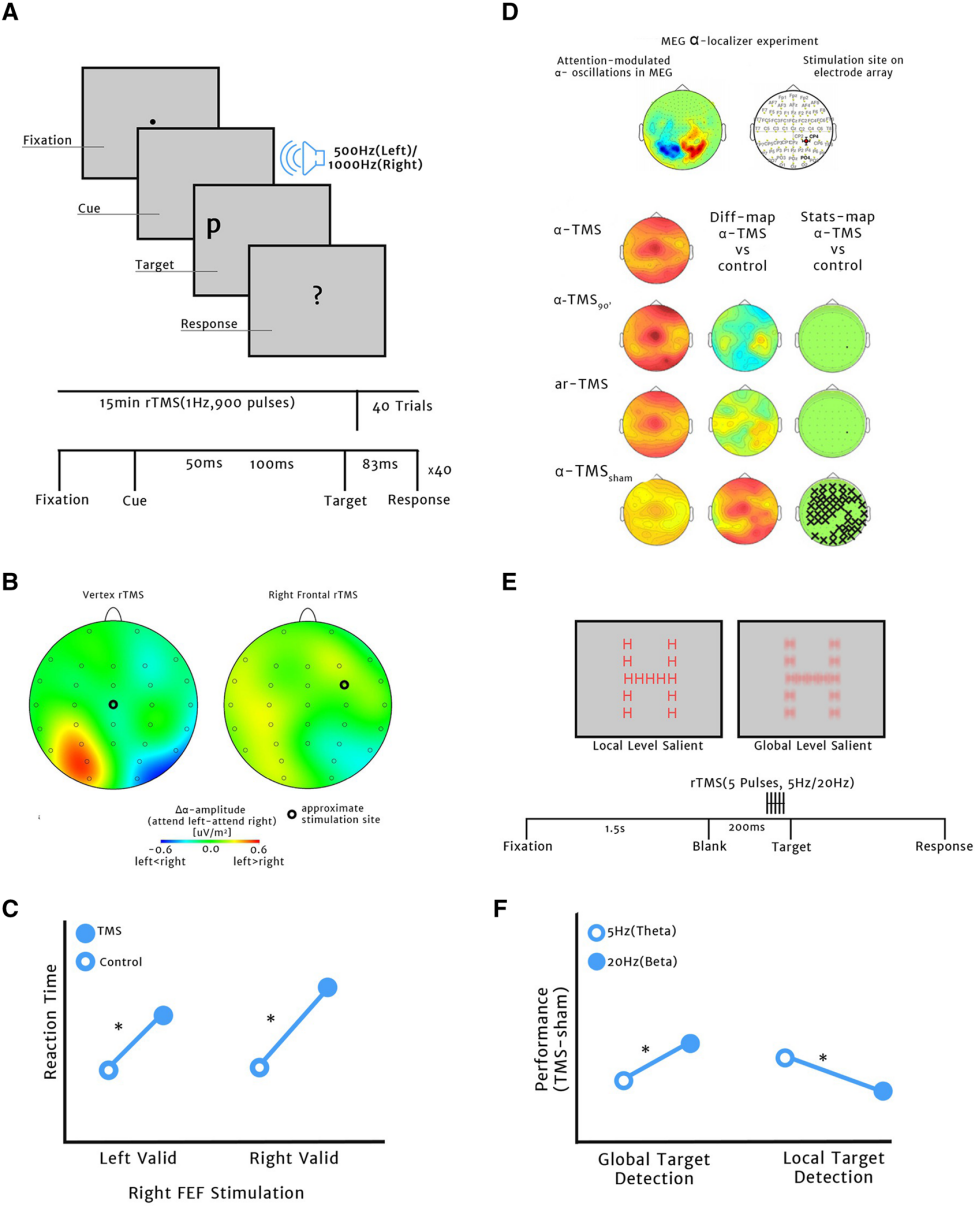
TMS investigations into the role of oscillations in attention. **a** Concurrent TMS-EEG to investigate the causal role of right FEF alpha oscillations in attention (Sauseng et al.^114^). (Top) Task structure. Following fixation an auditory cue (500 or 1000 Hz frequency) indicated the left or right hemifield for attention. After a variable interval of 600–800 ms a target letter, either a “p” or a “q”, was presented on the same or opposite hemifield. Subjects had to identify and report the target letter. (Bottom) Timeline of the experiment. rTMS at 1 Hz was delivered over the right FEF for 15 min (900 pulses), followed by experiment session with concurrent EEG recording. **b** EEG alpha amplitude map (topographic plots). The hemisphere specific alpha synchronization and desynchronization patterns observed normally during attention for the control (left panel) are nearly abolished following right FEF rTMS (right panel). **c** Behavioral effects of rTMS. On valid trials (on both left and right sides) reaction times of subjects who underwent FEF TMS increased compared to those who underwent control (vertex) TMS**. d** Rhythmic TMS entrainment of parietal alpha oscillations (Thut et al.^115^). (Top) Identification of parietal alpha generating sites and alpha frequencies in was done through MEG for individual subjects. TMS at the particular alpha frequency was then applied over this alpha generation site in a manner phase locked to the subject’s inherent parietal alpha rhythm. (Bottom, left column) MEG alpha amplitude maps following alpha TMS phased locked to inherent alpha (top row), alpha TMS with 90° tilt of the coil from the previous orientation (second row), arrhythmic TMS (third row) or sham TMS on a different site (bottom row). Of these, only phase-locked alpha TMS produced significant entrainment. (Middle column) Difference in alpha power for each stimulation condition compared to oscillation phase-locked alpha TMS. (Right column) Statistical map showing significant differences in alpha power. **e** Effects of beta and theta frequency entrainment in the parietal cortex (Romei et al.^119^). (Top) Task structure. Subjects detected the presence of a target letter (“H”) from Navon letter stimuli. Two conditions were tested with the letters sharp (left) or blurred (right). The stimuli could be either congruent—a global H comprised of local H–s—or incongruent (different local and global letter forms). Blurring rendered global detection easier, particularly for incongruent stimuli. (Bottom) Timeline of a representative trial. rTMS was delivered as 5 rhythmic pulses at theta (5 Hz) or beta (20 Hz) frequencies over the right parietal cortex before stimulus display, with the last pulse coinciding with stimulus onset. **f** Effects of entrainment on behavioral performance. Global detection was facilitated with beta frequency stimulation, whereas local detection was facilitated at theta frequencies; both effects were observed for incongruent stimuli.

In the first category are studies that applied TMS to probe the causal role ongoing natural oscillations by disrupting them. For instance, Dugue et al. applied double-pulse TMS over early visual areas during a conjunction search task. They showed that successful target detection depended on the phase of the ongoing alpha oscillation visual areas. Moreover, TMS disrupted search activity in a periodic fashion at a 6 Hz (theta-band) frequency.^39^ This suggests that visual search has a periodic component that may be mediated by particular brain oscillations. Similar theta-band periodicity for attentional reorienting was demonstrated by Dugue et al. using double-pulse TMS over the occipital (V1/V2) regions with a top-down, cued orientation discrimination task. They calculated the difference in discrimination performance between trials in which the target coding side versus distractor coding side were stimulated, and observed a 5 Hz modulation of this performance difference, but on invalidly cued trials alone.^116^ Other studies applied TMS over the FEF to disrupt ongoing oscillations. When cTBS was applied to inhibit FEF activity in a covert attention task, alpha-frequency modulation over contralateral visual cortex was diminished. Moreover, gamma power over the left FEF increased upon stimulation of right FEF.^117^ Sauseng et al. found that rTMS (1 Hz) over the right FEF, while subjects performed a centrally cued attention task disrupted the pattern of alpha power modulation (ipsilateral increase, and contralateral decrease) normally observed during attention tasks, and concurrently slowed response times during validly cued trials^114^ (Fig. 4a–c). Herring et al. used single-pulse TMS over the left visual cortex to elicit a TMS-locked alpha oscillation like response, and showed that this alpha-like response was suppressed during the performance of a visual attention task. The extent of attentional suppression of this TMS-evoked alpha like response could be predicted by the extent to which visual attention suppressed spontaneous alpha in the same region.^118^ These studies suggest that top-down control of visual cortex activity by the FEF during attention causally involves oscillations at alpha and gamma frequencies, and disrupting these oscillations could have direct effects on behavior.

In the second category are studies that applied patterned TMS for the rhythmic entrainment of oscillations, to study the role of these oscillations in attention. Entrainment is the process by which an external source undergoing periodic oscillations gradually synchronizes the phase of the ongoing neural oscillations to its rhythm. The external source, if strong enough, can also induce neural oscillations at a population level, which may subsequently influence behavior.

Studies that applied rhythmic entrainment can be, again, classified into two sub-categories. The first sub-category studied the effects of rhythmic entrainment in attention-related brain areas on sensory processing. For example, entrainment of the parietal cortex in theta and beta (15–30 Hz) frequencies facilitated detection of global and local features of the stimulus, respectively^119^ (Fig. 4e–f). Chanes et al. entrained gamma (50 Hz) and high-beta (30 Hz) oscillations in the right FEF using rTMS during a task involving spatial localization of a near threshold target. Their results suggested that beta entrainment specifically improved sensitivity of detection, while gamma entrainment specifically influenced response bias. Arrhythmic TMS, with the same number of pulses, did not show any of these behavioral effects.^120^ The second sub-category directly tested the effects of rhythmic entrainment in the context of attention tasks. For instance, Thut et al. used TMS at alpha frequencies to entrain alpha oscillations over parietal cortex; for each individual, stimulation frequency was tailored to the frequency of natural alpha oscillations as measured with MEG. Entrainment occurred only when the pre-TMS alpha oscillation and rhythmic TMS were aligned in phase^115^ (Fig. 4d).

In summary, TMS has offered critical insights into the mechanistic contribution of various brain regions to top-down and bottom-up attention, although a vast majority of the insights have been confined to the fronto-parietal cortex. These studies add to a growing body of literature suggesting that the effects of TMS depend crucially upon timing, hemispheric location, number of pulses and behavioral context (e.g., validly versus invalidly cued trials). Finally, TMS studies are also beginning to provide a clearer picture of the mechanistic role of brain rhythms in cognitive processing, and additional TMS studies are needed to investigate the role of specific brain rhythms in top-down versus bottom-up attention.

## Emerging Combinatorial Paradigms

We have, thus far, described conventional applications of TMS in attention, viz., for perturbing activity in specific brain areas and measuring effects on behavioral metrics. Emerging approaches that combine TMS with quantitative psychophysics or neuroimaging enable achieving more precise insights into the neural mechanisms of attention. We briefly discuss these new combinatorial paradigms here, with key implications for unraveling mechanisms of top-down and bottom-up attention.

Psychophysical models provide a parsimonious mapping between latent neural processes and behavior; combining TMS with psychophysical modeling, involves estimating model parameters from behavior with and without application of TMS.^121^ These model-based approaches have the potential to provide key mechanistic insights into neural processes underlying the behavioral effects of TMS.^122^^–^ 124 Such a modeling approach is particularly relevant for attention. For instance, recent quantitative psychophysical models have shown that that attention is not a unitary phenomenon.^125^^–^ 127 Attention (either top-down or bottom-up) may enhance perceptual performance through the operation of at least one (or both) of two component mechanisms. The first mechanism involves enhancing “sensitivity”, i.e., enhancing sensory processing of the attended target stimulus, at the expense of processing unattended distractors. The second mechanism involves enhancing choice “bias”, i.e., providing relatively greater weightage to the target stimulus in the downstream decision process, and filtering out distractors. Conventional signal detection theory models permit quantifying sensitivity and bias in simple perceptual tasks, but are insufficient to estimate sensitivity and bias in attention tasks. A recently developed psychophysical framework, the m-ADC framework, enables quantifying sensitivity and bias in attention tasks. This framework could be used, in conjunction with TMS, to identify the specific neural bases of sensitivity or bias control during top-down or bottom-up attention tasks.^125^

Transcranial electrical stimulation protocols typically produce long lasting effects (minutes to hours) on brain activity; concurrent TMS-tES is increasingly applied for investigating neural mechanisms of these tES effects. In such approaches, tES is used to modulate the underlying activity of a region of interest over long timescales (minutes to hours), and TMS (e.g., single-pulse or paired-pulse protocols) is used to transiently probe and quantify these modulatory effects. Because a single pulse of TMS over motor cortex readily evokes a ready readout of muscle excitability, in the form of the motor-evoked potential (MEP), such approaches have been extensively used to investigate motor cortex function, including investigations of corticocortical connectivity and corticospinal excitability.^128^^,^ 129 For instance, Feurra et al. applied 10 min of anodal tDCS stimulation of the right parietal cortex during a motor imagery and observation task. They showed that tDCS enhanced MEPs, corresponding to an increase in excitability, of the ipsilateral motor cortex during the motor imagery task.^129^ More recently, Nowak et al. employed a combined TMS-tACS paradigm to illustrate how gamma frequency tACS over the motor cortex, enhanced intracortical inhibition assessed by paired-pulse TMS stimulation.^130^ While such combinatorial stimulation paradigms have not yet found extensive use in the study of attention, combined TMS-tACS paradigms may help identify key mechanisms by which specific brain rhythms, as induced by tACS, alter neural excitability, as probed by TMS, during top-down or bottom-up attention tasks.

Electroencephalographic (EEG) recordings reveal the dynamics of neuroelectric processes occurring at fine timescales (few milliseconds); concurrent TMS-EEG protocols provide a sensitive readout of the effects of neurostimulation. Even when behavioral effects are not readily apparent, TMS produces systematic effects on the EEG.^37^ TMS effects on sensory or motor components of the event-related potential (ERP) are well studied; these effects vary with the site of stimulation.^131^^–^ 135 Typically, low-frequency (<1 Hz) rhythmic TMS enhances ERP amplitudes and decreases ERP latencies; high-frequency (>5 Hz) TMS produces converse effects. Rhythmic stimulation protocols, such as cTBS, have been shown to produce large (~30%) and sustained (15–70 min) decreases in ERP amplitudes, thereby providing a neural basis for the effects of these protocols on behavior.^37^ A few concurrent TMS-EEG studies have investigated the roles of the prefrontal and parietal cortex in the context of attention. Captosto et al. explored the effects of applying a single rTMS train of 20 Hz for 150 ms on the right FEF and IPS of subjects performing a stimulus detection task, in the anticipatory period at attention cue onset and before target onset.^136^ The authors observed that anticipatory alpha rhythms in the occipital lobe were desynchronized and identification of the target was impaired. Similarly, ERP coupled rTMS (5 pulses at 10 Hz) applied to the right FEF between cue and target onset in a top-down attention task produced a negative deflection both in cue-evoked and target-evoked ERPs.^137^ Finally, single-pulse TMS of the right PPC during a conjunction task 100 ms after array onset produced a delay in reaction times that corresponded to the disruption of a 250-300 ms component of a visual ERP.^99^ EEG-TMS provides a powerful tool for measuring the electrophysiological correlates of stimulating particular brain areas, and may be crucial for temporally precise probing of the mechanistic involvement of fast neural processes (e.g., ERPs or neural oscillations) in top-down and bottom-up attention tasks.

Finally, combining TMS with fMRI provides an unprecedented opportunity for precise stimulation of functionally defined brain regions, as well as for visualizing the effects of TMS on neural activity across the whole brain.^138^ In an fMRI-informed TMS study Sack et al.^139^ acquired fMRI, while subjects performed a target detection task, based on feature conjunctions, followed by TMS (Fig. 3a–c). Left and right IPS loci that showed activation during task performance were then targeted for rTMS (600 pulses at 1 Hz). rTMS disrupted performance by increasing reaction times compared to control conditions, linking IPS neural activity to top-down visuospatial detection.^139^ Concurrent TMS-fMRI permits investigating local effects of TMS at the site of stimulation as well as global effects in areas connected by long-range projection fibers to the area being stimulated. Several studies have employed fMRI to measure alterations of brain activity in sensory areas following TMS of attention-related areas. Ruff et al. applied rTMS (5 pulses at 10 Hz) to the right FEF and observed an increase in the BOLD response of retinotopic visual areas; the stimulation also produced an increase in the apparent (perceived) contrast of peripheral stimuli.^140^ Blankenburg et al. applied 10 Hz rTMS (5 pulses) at the PPC, during a top-down discrimination task, and observed that certain occipito-parietal areas that showed higher activation during attention to contralateral than ipsilateral stimuli in control conditions underwent an increase in the magnitude of this differential activation upon stimulation. Behavioral performance, though, remained unaltered.^141^ TMS-fMRI has also provided putative evidence for dissociable roles of the PFC and PPC in attention to motion features^142^: TMS over the IPS, but not FEF, produced a reduced BOLD response to motion stimuli in area MT.^142^

As demonstrated by these findings, emerging combinatorial paradigms are likely to be invaluable in the search for specific neural mechanisms of top-down versus bottom-up attention. Nevertheless, significant technical challenges must be overcome before these paradigms can be widely adopted, as discussed in the next section.

## Challenges

Despite the promise of TMS, and combinatorial TMS-imaging paradigms, several key challenges, both with the application of these techniques and the interpretation of findings, remain. We discuss these here along with recent technological advances that have provided a starting point for addressing these challenges.

First, TMS effects are variable across individuals. The physiological effects of TMS depend heavily on the precise structural topography of the brain region being stimulated, including the depth and angle of gyral folds relative to the orientation of the TMS coil, the density and orientation of axons and cell bodies, the size of and inter-connectivity within the brain region, and the like. Unsurprisingly, significant variability has been reported in the size, duration and even direction of TMS’s behavioral effects across individuals, likely due to inter-individual differences in brain structure.^31^ This limits reproducibility across studies, especially those testing small cohorts of individuals for subtle behavioral effects, as is common in attention tasks. Conventionally, the positioning of TMS coils is guided by sophisticated neuro-navigation algorithms that rely on high resolution structural MRI scans to align the TMS coil at particular positions and orientations to target specific brain areas in each subject. Advances in neuro-navigation algorithms permit constructing more sophisticated head models to guide more precise positioning of the TMS coil and to get more accurate estimates of the spread of stimulation across adjacent brain areas.^13^ Moreover, recent advances in diffusion imaging techniques (dMRI) permit measuring inter-individual differences in brain structural connectivity that might underlie differences in TMS effects. For instance, a recent study showed that differences in connectivity across the corpus callosum were predictive of inter-individual differences in behavioral accuracy, presumably due to differences in interhemispheric coordination.^143^ In addition, TMS effects are also highly sensitive to stimulation parameters. For instance, oscillatory entrainment in parietal or frontal areas does not occur reliably even with minor differences in the stimulation protocol in terms of stimulation site, alignment of the frequency and phase of the applied TMS with respect to inherent oscillations and the like, again potentially due also to inter-individual differences in physiology.^115^^,^ 128 To partly address this source of variability, recent studies have applied rhythmic neurostimulation by matching stimulation frequencies for each subject to her/his frequency of natural brain oscillations, as recorded by EEG/MEG.^115^

Second, it is increasingly clear that the effects of TMS also vary depending on the timing of stimulation relative to the underlying neural state. Converging evidence indicates that stimulation immediately prior to the onset of stimulus facilitates behavioral performance, while stimulation during stimulus presentation usually disrupts performance. For instance, stimulation preceding stimulus onset improved object naming latency and target detection efficiency 93 ^,^ 144, whereas stimulation after target onset in motion discrimination and target discrimination tasks reduced discrimination accuracy.^38^^,^ 103 ^,^ 145 These results have been explained as follows: if TMS were to enhance the excitability of neural populations in the quiescent state, TMS delivered just before the stimulus may enhance neural excitability, and improve stimulus processing thereby facilitating behavioral performance. On the other hand, during an ongoing task, when task-relevant neural populations are already highly active (at ceiling), TMS may increase the excitability of task-inhibitory or task-irrelevant neural populations thereby disrupting processing and behavioral performance. These state-dependent effects manifest in other forms as well. For instance, Silvanto et al. showed that TMS on FEF produced phosphenes with the color of the stimuli of a previous color adaptation task, when the stimulation followed the adaptation task.^146^ This state-dependent effect of TMS can be utilized for selective analysis of neural populations. By preventing the activation of neurons that encode specific stimulus features, for example, with sensory adaptation, TMS’s effects on the non-adapted populations of neurons can be selectively investigated.^146^ These approaches could provide essential insights into the role of specific neural populations that encode particular features (e.g., orientation or color) in mediating the effects of top-down and bottom-up attention.

Third, although TMS has been widely used for stimulating cortical tissue (2–3 cm) from the scalp surface) with conventional coils, it is not ideal for stimulating deeper, sub-cortical structures. Even in cases where higher stimulation strengths have been used to stimulate deeper structures (e.g., at 120% of motor threshold^147^), collateral activation of neural tissue superficial to these structures is inevitable. Nevertheless, gaining a full understanding of cognitive processes, like attention, demands the ability to stimulate deeper brain structures like the superior colliculus and basal ganglia. To achieve stimulation of deeper brain structures, recent studies have employed an indirect approach—by stimulating cortical connections that share strong anatomical connectivity with these sub-cortical areas, and confirming their indirect activation with concurrent fMRI. For example, Wang et al. applied high-frequency rTMS to the lateral parietal cortex, part of a cortical-hippocampal network and demonstrated enhanced functional connectivity within this network as well as improved associative memory performance.^148^ An important point to note with this approach is that it is difficult to dissociate the effects of indirect modulation of the sub-cortical structure from those of the direct modulation of the cortical region, and the results must be interpreted as arising from activation of a distributed network. Recent improvements in hardware seek to achieve focal stimulation of deeper brain areas through advanced coil designs (e.g., H1/H2 coils).^149^ Although these are being developed primarily for therapeutic purposes, once commercialized, these have the potential to provide important insights into the role of key sub-cortical brain structures in top-down and bottom-up attention.

Fourth, although emerging combinatorial paradigms permit more precise evaluation of the neural mechanisms of TMS; these also come with important technical challenges. For example, it remains a significant challenge to analyze EEG signals recorded concurrently with TMS, because of the large TMS-induced artifacts in the EEG signal (but see^150^). Similarly, concurrent TMS-fMRI involves taking into consideration important safety issues associated with performing TMS inside MR scanner: the strong magnetic field and switching gradients within the MRI scanner bore can cause heating up and unsafe temperature rise in the TMS coil. In addition, the large TMS magnetic field gradients can induce significant artifacts in fMRI recordings.^151^^,^ 152 These challenges need to be overcome before combinatorial paradigms can find widespread application.

Fifth, despite decades of study, the precise neurophysiological mechanisms of TMS remain unclear. Animal model studies are constrained due to the necessity of scaling the coil size correspondingly to the head size to maintain stimulation efficiency^153^, although efforts are being made to overcome some of these challenges.^154^ Additionally, advances in computational modeling of stimulation effects and integration of TMS with functional imaging and spectroscopy may provide greater insights into the precise mechanisms of stimulation.^155^

Finally, TMS faces a key challenge, one also shared by other brain stimulation techniques, in terms of interpreting the effects of stimulation. The conventional approach to neuroscience emphasizes understanding the functional role of particular neural populations in specific brain regions. Hence, neurostimulation techniques have traditionally sought to study the effect of spatially focal stimulation of particular brain regions (e.g., with TMS) or of specific neural groups (e.g., with optogenetics). However, this view is changing rapidly: it is increasingly clear that cognitive processes, like attention, require the coordinated activity of neural populations across multiple brain regions. Thus, behavioral effects of TMS, or indeed, of any neurostimulation technique, are likely to arise not from altered activity of an isolated neural population, but rather as a consequence of concurrent changes in several connected neural populations across brain regions acting together as functionally coupled networks. Taking these network effects into consideration is crucial for fully interpreting the results of stimulation studies, particularly those that seek to understand the neural underpinnings of complex cognitive phenomena. Recent work in network neuroscience, based on network control theory, has begun to address key challenges produced by this emerging perspective of brain function.^156^

## Conclusions and Future Directions

While invasive neurostimulation studies in non-human primates, and other mammals, have provided key insights into attention mechanisms, recent advancements in non-invasive technologies, and combinatorial paradigms, have opened up new frontiers for testing these mechanisms in the human brain. Humans can be readily trained to perform complex attentional tasks, and human TMS studies carry the advantage of being able to evaluate subtle differences between top-down and bottom-up attention control using sophisticated task designs. Moreover, mechanisms of attention control, as discovered with human TMS studies, can form the basis for constructing further, detailed hypotheses of how these mechanisms operate at the level of neural circuits. These models may then be tested with cellular and circuit-level manipulations (e.g., optogenetics) in non-human primate and other animal models.

Of particular interest is the role of fronto-parietal areas in mediating top-down and bottom-up attention. There is active research and debate on the distinct involvement of these areas and their timing of activation during the two modes of attention.^106^^,^ 109 ^,^ 137 Receptive field sizes of the frontal and parietal regions are much larger (over 100°) as compared to early sensory coding areas (e.g., V1—0.5° to 2°). The regions also have extensive reciprocal connections with many upstream executive control regions, such as DLPFC and downstream sensory regions, such as MT, V4 and V1.^41^^,^ 85 This allows these regions to accumulate information across the visual field to form a bottom-up ‘saliency map’, and then integrate this with top-down goals to form a ‘priority map’ of the environment.^157^^,^ 158 How the saliency and priority maps are combined to determine the next target for the allocation of visual attention, either top-down or bottom-up, remains an active area of research,^72^ and TMS can shed light on the distinct involvement of the frontal and parietal regions in computing and integrating these maps.

In this review, we have focused primarily on mechanisms of top-down and bottom-up visual spatial attention. However, these two modes of attention control certainly operate for other forms of attention (e.g., attention to features or objects) and other sensory modalities (e.g., audition). MEG recordings in humans have shown that the inferior frontal gyrus is critically important, and could mediate selection of relevant stimuli during object-based attention.^159^ Non-invasive neurostimulation will be key to unraveling whether brain regions and neural mechanisms for object-based or auditory attention control are shared with those for top-down and bottom-up visuospatial attention.^160^

TMS is also particularly important in terms of its translational potential. TMS has found wide use in therapeutic interventions for treating neuropsychological disorders like drug resistant epilepsy, Parkinson’s disease and major depressive disorder. Low-frequency rTMS applied for 15–30 min can be used to suppress seizure generating excitatory activity in epileptic foci as well as abate extant seizure activity. rTMS has also been used for alleviating gamma oscillation deficits in autism and schizophrenia.^161^^,^ 162 rTMS sessions in rats were shown to induce plastic molecular changes—including changes in the levels c-fos, glial fibrillary acidic protein (GFAP), brain derived neurotrophic factor (BDNF), cholecystokinin or corticotropin (ACTH)—resembling the effects of antidepressants or electroconvulsive therapy interventions.^26^^,^ 163 Novel TMS techniques to focally stimulate particular deep brain regions are being developed for medical use and clinical trials. Together with emerging imaging technologies, TMS can pave the way toward a more complete understanding of the neural basis of selective attention, as well as other cognitive phenomena, both in health and in disease.
